# Impact of Glucocorticoid Excess on Glucose Tolerance: Clinical and Preclinical Evidence

**DOI:** 10.3390/metabo6030024

**Published:** 2016-08-03

**Authors:** Aoibhe M. Pasieka, Alex Rafacho

**Affiliations:** 1 School of Kinesiology and Health Science, Muscle Health Research Centre, York University, Toronto, ON M3J 1P3, Canada; pasieka1@yorku.ca; 2 Department of Physiological Sciences, Center of Biological Sciences, Federal University of Santa Catarina, Florianópolis 88040-900, Brazil

**Keywords:** dexamethasone, glucose homeostasis, glucose tolerance, insulin sensitivity, insulin signaling, outcomes, side-effects, peripheral tissues, prednisolone

## Abstract

Glucocorticoids (GCs) are steroid hormones that exert important physiological actions on metabolism. Given that GCs also exert potent immunosuppressive and anti-inflammatory actions, synthetic GCs such as prednisolone and dexamethasone were developed for the treatment of autoimmune- and inflammatory-related diseases. The synthetic GCs are undoubtedly efficient in terms of their therapeutic effects, but are accompanied by significant adverse effects on metabolism, specifically glucose metabolism. Glucose intolerance and reductions in insulin sensitivity are among the major concerns related to GC metabolic side effects, which may ultimately progress to type 2 diabetes *mellitus*. A number of pre-clinical and clinical studies have aimed to understand the repercussions of GCs on glucose metabolism and the possible mechanisms of GC action. This review intends to summarize the main alterations that occur in liver, skeletal muscle, adipose tissue, and pancreatic islets in the context of GC-induced glucose intolerance. For this, both experimental (animals) and clinical studies were selected and, whenever possible, the main cellular mechanisms involved in such GC-side effects were discussed.

## 1. Introduction

Glucocorticoid (GC) secretion is primarily regulated by the hypothalamic-pituitary-adrenal axis. Elevations in endogenous GCs (cortisol in humans, corticosterone in rodents) result from conditions that represent a threat to metabolic homeostasis such as hypoglycemia, trauma, infections, intensive heat or cold, as well as general stressful situations [1]. Working together with other counter-regulatory hormones (e.g., adrenaline, glucagon), elevations in endogenous GC lead to reduced peripheral insulin sensitivity, which in turns diminishes the peripheral glucose disposal providing organism with glucose [1,2]. Despite the capacity to increase blood glucose levels, a theme that will be focused in the present review, GCs also exert (i) potent immunosuppressive and anti-inflammatory actions; (ii) modulate the control of lipid and protein metabolism; (iii) modulate the central nervous system and behavioral components; and (iv) have important actions in bone and calcium metabolism [2]. Their central and peripheral actions are well characterized in pathologic conditions of excessive GC secretion, such as in Cushing’s Syndrome, or during GC deficiency, as in Addison’s disease [1,2].

Multiple GC actions, particularly their anti-inflammatory properties, led to development of several potent synthetic analogues with a higher half-life and potency, as well as more selective actions relative to the endogenous cortisol. Thus, synthetic GCs (e.g., prednisolone (PRED) and dexamethasone (DEX)) are pharmacological approaches mainly devoted to the treatment of acute and/or chronic inflammatory- and autoimmune-related diseases/episodes [3]. GC actions are cell- and tissue-specific and vary depending on many aspects including dosage, duration of treatment (short- or long-term), previous individual susceptibility (e.g., glucose intolerance or reduced insulin sensitivity), and the species evaluated (e.g., human, rodents) [4,5]. Since the actions of GCs may be both direct and indirect, it is difficult to obtain a consensual comprehension of their actions.

When in excess, independent of treatment duration, GCs generally promote adverse metabolic effects. For instance, GC treatments may decrease the insulin-suppressive effects on hepatic glucose output (HGO) [6,7,8,9,10], reduce the insulin-stimulated glucose uptake in the skeletal muscle and adipose tissue [11,12,13,14], as well as impair the disposition index (the matching insulin secretion determined by a given peripheral insulin sensitivity) [15,16,17,18,19,20]. In the following sections we will describe the main preclinical and clinical experimental research on GC actions related to glucose metabolism, especially that of DEX and PRED, emphasizing the foremost alterations occurring in the liver, skeletal muscles, white adipose tissue and pancreatic islets. In this review, we summarize a number of studies’ outcomes demonstrating how GCs in excess disrupt glucose homeostasis and promote typical glucose intolerance, which may predispose patients undergoing GC therapies to develop overall type 2 diabetes *mellitus* (T2DM).

## 2. Effects of GC Excess in the Liver: Contribution of Increased HGO to GC-Induced Glucose Intolerance

**Figure 1:**
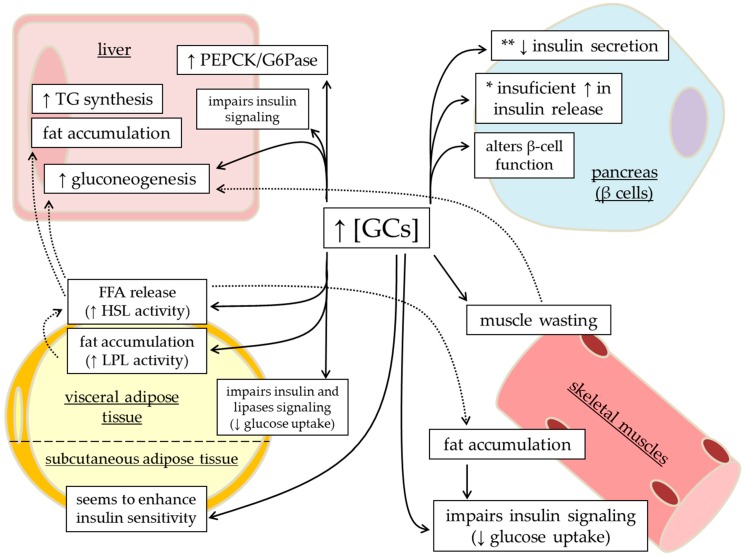
Adverse actions of glucocorticoids (GCs) on peripheral tissues involved in the control of glucose homeostasis. Excess or prolonged GC treatment may disrupt glucose homeostasis by interfering with several metabolic-related tissues. In visceral adipose tissue, GCs elevate LPL activity, leading to fat accumulation at this fat site; while at the same time exhibiting increased HSL activity, resulting in increased lipid (FFA and glycerol) release, supplying for hepatic triacylglycerol synthesis, fat accumulation and gluconeogenesis, and also in intramuscular fat accumulation. These steroids may also affect insulin signaling in adipose tissue. GCs impair insulin-stimulated glucose uptake in skeletal muscles and induce muscle wasting, which, in turn, provides gluconeogenesis substrates. In the liver, GCs have a negative effect on rate-limiting enzymes controlled by insulin. Finally, * GC in excess may also lead to an insulin hypersecretion that may not be sufficient to match with the disposition index, which means relative increase in islet function or ** cause insulinopenia depending on previous individual’s susceptibility (read Section 5 for more details). Continuous line means direct effect, while dotted lines means indirect action. **FFA**, free fatty acids; **G6Pase**, glucose-6-phospatase; **HSL**, hormone-sensitive lipase; **LPL**, lipoprotein lipase; **PEPCK**, phophoenolpyruvate carboxykinase; **TG**, triacylglycerol. Modified from Rafacho et al. [30].

GCs are well known to induce alterations in glucose homeostasis and impairments in glucose tolerance are amongst the common adverse effects during GC therapies [2], as well as in patients with Cushing’s syndrome [21]. Abnormal upregulation of hepatic gluconeogenesis plays a major role in the pathophysiologic process of increased hepatic glucose production (HGP) in conditions of insulin resistance related with GC excess. In a physiologic context, GCs and insulin act in opposing directions, affecting the expression of the two key gluconeogenic enzymes, phosphoenolpyruvate-carboxykinase (PEPCK) and glucose-6-phosphatase (G6Pase). GCs are known to induce gluconeogenesis by stimulating the expression of PEPCK and G6Pase gene (Figure 1), while insulin decreases this process through inhibition of PEPCK and G6Pase gene expression (for a comprehensive review, see [22]). Despite glucose tolerance impairments with excess GCs, the presence of endogenous GCs is necessary for an adequate hepatic glucose homeostasis [23].

A number of preclinical and clinical studies have demonstrated that GC administration, at high doses and/or chronic periods (days to weeks), promotes a dysregulation in hepatic glucose metabolism that is directly related to the reduction of the insulin action in the liver, which ultimately means hepatic GC-induced insulin resistance (IR) [6,17,19,24,25,26,27] (Table 1). Olefsky and collaborators [28] performed one of the first experiments that demonstrated the negative impact of GCs on insulin binding to its receptor in isolated hepatocytes. The authors found a dose-dependent effect of DEX (1.5 mg/kg or 0.125 mg/kg for 6 days), in that the insulin binding in GC-treated rats was only 30%–50% of controls. When the lower dose was maintained for a more prolonged period of 21 days, the insulin binding was still kept at 55% of control values, suggesting that GC action on this parameter is not transient, but rather continuous during longer periods of GC treatment.

Subsequently, a number of clinical studies demonstrating the effects of acute GC administration revealed an increase in blood glucose levels and/or blood glucose area-under-curve values during an oral glucose tolerance test (oGTT) after treatment with cortisol [29] or DEX [15,24]. Interestingly, in both cases, the hepatic glucose production (HGP) was not increased in these individuals, thus, the elevation in blood glucose concentration seemed to result from a decrease in the peripheral glucose uptake and/or glucose clearance [15,29], as well as from an increase in the hepatic G6Pase activity [24]. Two out of these three studies showed that these alterations occurred with no indication of altered peripheral insulin sensitivity [15,29]. Another clinical study, however, demonstrated the ability of short-term DEX treatment to induce an increase in HGP, without impairment in the whole-body glucose uptake [9]. These discrepancies (see in Table 1) may vary according to the protocol design (dosage and duration of treatment, gender, whether cortisol, PRED or DEX), as well as the individual differences in susceptibility amongst subjects. In this context, hepatic sensitivity to GCs was explored in volunteers with high or low responsiveness to insulin [25]. The former group developed only a slight alteration in glucose homeostasis and exhibited an appropriate insulin hypersecretion (normal disposition index). However, the low insulin responders had higher fasting glycemia and became more glucose intolerant after 7.5 mg or 15 mg of DEX over 48 h. In fact, low-insulin responders had augmented HGP, HGO and glucose cycling (HGO minus HGP) indicating that the relative β-cell failure is a determinant for the disruption of liver glucose homeostasis with GC excess [25].

In subsequent studies, it was shown that a low dose of daily oral PRED administration, 6 mg for 7–10 days [34] or 7.5 mg for 14 days [10] also induced a deterioration in glucose homeostasis, although to a lower magnitude than imposed by the high PRED dose (30 mg) [10]. After two weeks of daily 7.5 mg PRED, healthy men exhibited a slight increase in fasting blood glucose values (from 88–92 mg/dL) that was associated with lowered insulin potency to suppress HGP, however, no evidence of reduced peripheral glucose uptake was detected during an insulin infusion in subjects treated with 7.5 mg PRED. Similarly, Petersons and colleagues [34] demonstrated that 7–10 days of PRED treatment (6 mg, daily) resulted in an increase in basal endogenous glucose production (EGP) and reduced insulin suppression on EGP. In this study, authors showed that the chronic use of low PRED doses (4–10 mg/day) might have a more mild effect on insulin-stimulated glucose uptake than short-term treatments (6 mg/day) [34]. Even though prolonged exposure to low doses of GC negatively impacts intermediate glucose metabolism (i.e., hepatic insulin-receptor binding), most of the clinical studies with healthy individuals did not result in fasting glycemia greater than 126 mg/dL (7 mM) [9,17,19,31,35]. Although GC therapies must be used with caution, especially in susceptible individuals [36,37], it is important to note that the parameters that were altered in these individuals returned to baseline values after interruption of GC treatment [17].

The mechanisms by which GCs impair hepatic glucose homeostasis are not yet fully elucidated in humans and merit continuous investigation. Studies with laboratory animals have brought some mechanistic evidences. Rats treated with DEX for 7–10 consecutive days at 0.5 mg/kg body weight (*b.w.*) [8] or 1 mg/kg *b.w.* [6,7] develop enhanced HGO as demonstrated using perfused liver or glucose/insulin clamp techniques; corroborating with the prior clinical studies mentioned (Table 1). Insulin’s ability to suppress HGO is impaired in the liver of these rats, indicating that the insulin-signaling pathway is probably involved in the disrupted insulin response in the liver [8]. In a similar protocol (1 mg/kg, *b.w.*, for 5 days), DEX treatment provoked a decrease in insulin-induced insulin receptor and insulin receptor substrate (IRS)-1 tyrosine phosphorylation in the liver of rats. These animals also exhibited a reduction in insulin-stimulated phosphoinositide 3-kinase (Pi3K) activity in anti-IRS-1 immunoprecipitates from liver lysates, providing evidence for the involvement of GCs in the pathogenesis of insulin resistance (IR) at the cellular level [26]. Subsequent studies demonstrated that GCs may impact liver glucose metabolism independent of ligand binding to the GC receptor (GR) and GR dimerization [38]. By using a genetically engineered model with denominated GRdim (a mouse model unable to form GR dimerization, and thus, to interact adequately with GCs responsive elements (GREs)), authors showed that in GRdim mice the level of PRED-induced liver gene expression (1 mg/kg *b.w.*) was significantly decreased relative to wild-type, but was not completely absent. Interestingly, for a set of genes, implicated in cell cycle and apoptosis processes, induction by PRED was completely abrogated in GRdim mice. In contrast, glucose metabolism-related genes were still modestly upregulated in GRdim mice receiving PRED treatment [38]. Furthermore, activation of liver X receptors/retinoid X receptors (LXRs/RXRs) with the GW3965 ligand compound resulted in milder alterations in blood glucose homeostasis in DEX-treated rats (1.5 mg/kg *b.w.*). It also suppressed DEX-induced mRNA expression of hepatic G6Pase in rats, mice and human hepatoma HepG2 cells, whereas endogenous, unliganded LXRs were required for DEX-induced mRNA expression of PEPCK [27]. Both in vitro (hepatocytes) and in vivo (lean mice) studies demonstrated that mitogen-activated protein kinase phosphatase (MKP)-3 and forkhead box protein O (FOXO)-1 are downstream components of the adverse effects of DEX [39]. In this study the authors demonstrated that insulin-induced protein kinase B (PKB) Thr^308^ phosphorylation is significantly reduced in the liver of mice treated with DEX (15 mg/kg *b.w.* for 16 weeks) that parallels with IR and hepatic lipid accumulation. Such negative impacts of DEX treatment were prevented in MKP-3 deficient mice. Another component involved in the adverse GC side effects on glucose metabolism may be glucagon receptor. Treatment of adult male rats with glucagon receptor antagonist (des-His^1^-[Glu^9^]-glucagon (1–29) amide) abolished the increase in fed, and to a lesser extent, in fasting blood glucose caused by DEX treatment (1 mg/kg *b.w.*) [40].

**Figure 2:**
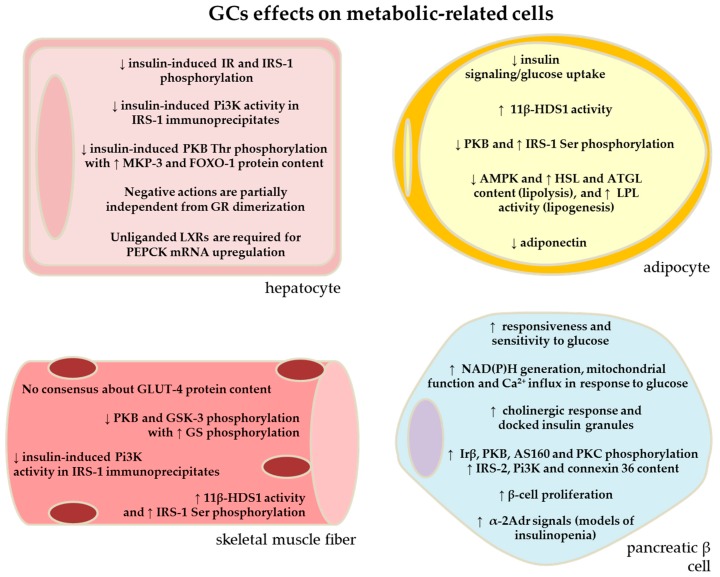
Cellular mechanisms involved with the glucocorticoid (GC) side effects. The main cellular components or pathways altered in metabolic-related cells from experimental models subjected to GC administration. Please, find abbreviations in the list of abbreviations.

This data, summarized in Figure 1 and Figure 2, reveals the complexity of mechanisms that intervene with the pattern of gene expression that are up- or down-regulated in liver from GC treated subjects and may account for the increased gluconeogenesis and subsequent glucose intolerance in these individuals. A constellation of factors including insulin signaling impairments, non-genomic GR effects, as well as the crosstalk with the receptor that responds to lipid-metabolites opens a field that requires further investigations.

## 3. Effects of GC Excess in the Skeletal Muscle: Contribution of Reduced Glucose Uptake to GC-Induced Glucose Intolerance

The negative impact of endogenous or exogenous GC excess on glucose homeostasis involves its ability to impair whole body glucose disposal [2]. Skeletal muscles play a pivotal role in this context considering that they represent the main site of insulin-mediated glucose disposal [41] and are commonly involved in the peripheral IR induced by the GCs excess; wherein insulin fails to effectively stimulate glucose uptake (Figure 1) [11,12,13].

Schonberg and colleagues [42] performed the initial studies focusing on the effects of DEX on muscle and demonstrated a positive effect of DEX on glucose uptake by examining the direct effects of DEX in L8 and L6E9 rat muscle cell lines. This data contradicted subsequent studies performed in rats treated with DEX that revealed a negative impact of GC treatment in the insulin-stimulated glucose uptake [7,8,12,13,43]. This negative impact of GC excess on peripheral glucose disposal was also demonstrated in healthy humans that were subjected to cortisol infusion [29], oral DEX administration (2 mg over 2 days) [11,14] or oral PRED administration (6 mg over 7–10 days) [34], which were associated with mild elevations in blood glucose [29] (Table 1).

The potential mechanisms by which GCs induce muscle IR have been reasonably well elucidated through the use of rodent models. It may involve muscle synthesis of epinephrine [44], which is known to exert similar actions as GCs on skeletal muscle, i.e., decrease glucose uptake and oxidation [11]. The contribution of glucose transporter (GLUT)-4, a protein recruited to the cell surface in response to insulin plays a major role in the stimulation of glucose transport into skeletal muscles in rats treated with DEX [7,43,45,46]. Studies have shown conflicting findings in regards to GLUT-4 protein content when examining the soleus and *extensor digitorum longus* during GC treatment. GLUT-4 protein content has been shown to both increase [45,46], or remain unaltered [7,43] after GC treatment. These discrepancies can be attributed to the variations between protocols (e.g., dose and duration of DEX administration as well the type of muscles investigated). A study conducted by Weinstein and collaborators [45] demonstrated that soleus muscles from rats treated with DEX for 2 days (0.8 mg/kg *b.w.*) decreased 2-[^3^H] deoxyglucose (2-[^3^H]DG) uptake at (i) basal and all concentrations of (ii) insulin and (iii) insulin-growth factor (IGF)-1 tested, and in response to (iv) hypoxia-stimulated 2-[^3^H]DG uptake. These and other complementary studies suggest that the inherent activity of plasma membrane bound GLUT-4 [6] and/or subcellular trafficking/recruitment to the cell surface [45,46] seems to be the possible mechanisms by which GCs impact on GLUT-4 signaling and ultimately impair insulin and non-insulin stimulated glucose uptake.

In parallel to GLUT-4 studies, it was shown that five days of DEX treatment, in normal rats, produced no alterations in the insulin-induced insulin receptor and IRS-1 tyrosine phosphorylation in muscles lysates [26]. However, the authors observed a significant reduction in insulin-stimulated Pi3K activity in anti-IRS-1 immunoprecipitates, illustrating the negative impact of GCs in the pathogenesis of IR at the cellular level. Studies led by Jensen’s group elucidated several aspects of insulin signaling in muscles from DEX-treated rats [12,13,47]. It was found that 11–12 days of DEX treatment resulted in a diminished ability of insulin to stimulate glucose uptake, glycogen synthesis and glycogen synthase (GS) fractional activity either in soleus and epithroclearis muscles [12]. In addition, insulin-induced dephosphorylation of GS was inhibited in soleus muscles from DEX-treated rats. These impairments were accompanied by reduced (i) PKB Ser^473^ and Thr^308^ phosphorylation; (ii) glycogen-synthase kinase (GSK)-3 β Ser^9^ protein phosphorylation, and increased (iii) GS Ser^7^, GS Ser^641^ and GS Ser^645,649,653,657^ protein phosphorylation both in the soleus and in epitrochlearis muscles in response to insulin [12,13]. In addition, selective pharmacological inhibition of GSK-3 with the CHIR-37 compound improved the ability of insulin to activate GS in skeletal soleus muscle from DEX-treated rats, however, it did not re-establish the reduced insulin-stimulated glucose uptake in muscles from DEX-treated rats. This ruled out, at least in part, the crucial role for GSK-3 in this process [12]. There is evidence to suggest that the p85α regulatory subunit of the Pi3K [48] and that protein kinase C (PKC) [49] also play critical roles in the GC-induced reduction of insulin-stimulated glucose uptake and insulin response in C2C12 myotubes or rat soleus muscles, respectively.

Interestingly, Jensen’s group demonstrated that DEX treatment in rats (1.0 mg/kg *b.w.* DEX for 12 days) does not present significant impairments in glucose uptake during muscle contraction (compared to resting condition) in soleus or epitrochlearis muscles [47]. In the epitrochlearis, but not in soleus of DEX-treated rats, the presence of insulin during contraction augmented glucose uptake to similar levels to that of the controls. These results were paralleled with reduced GS protein phosphorylation at Ser^645,649,653,657^ in epitrochlearis muscle, but contraction did not normalize the decrease in PKB Ser^473^ and GSK-3 protein phosphorylation that is commonly observed in this DEX rat model [47]. These authors suggested that activation of protein phosphatase (PP)-1 could cause dephosphorylation of GS, and contraction could activate PP-1 in DEX-treated rats. Still in this context, 5′ adenosine monophosphate-activated kinase (AMPK)-α phosphorylation is known to be activated during contraction and could be the signal link for the PKB-independent mechanism of action that would explain GS protein dephosphorylation [47]. Surprisingly, the protein content of contraction-induced AMPK-α Thr^172^ phosphorylation was lower in epitrochlearis (but not in soleus) muscle from DEX-treated rats [47]. Although the precise mechanisms for this positive impact of contraction on insulin action in insulin-resistant muscles with DEX treatment are still unclear, regular physical activities and/or exercises should be considered as a potential stimulus for counterbalancing the cellular side effects of GCs on skeletal muscle insulin sensitivity [47].

The prereceptor enzyme 11β-hydroxysteroid dehydrogenase type 1 (11β-HSD1), an endo-lumenal enzyme that converts inactive cortisone (11-dehydrocorticosterone (11-DHC) in rodents) into active cortisol (corticosterone in rodents), is also involved in the negative actions of GCs on skeletal muscles. 11β-HSD1 is expressed in insulin-target tissues including liver, adipose, and muscles as well as modulating the tissue GC availability [50]. Consistent with the effects of DEX on C2C12 rodent skeletal myocytes, incubation of C2C12 cells with corticosterone caused a dose- and time-dependent reduction in total IRS-1 protein content and increase in IRS-1 Ser^307^ protein phosphorylation [51]. Incubation of C2C12 myocytes with 11-DHC reproduced similar IRS-1 data as that found with corticosterone. These defects in C2C12 cells were abolished when cells were incubated with the selective 11β-HSD1 inhibitor compound, A1 [51]. Administration of A2 (a selective 11β-HSD1 inhibitor of higher purity than A1) in KK mice, a mouse model of metabolic syndrome that exhibit enhanced IRS-1 Ser^307^ and diminished PKB Thr^308^ protein phosphorylation in skeletal muscle, markedly reduced IRS-1 Ser^307^ and increased PKB Thr^308^ protein phosphorylation [51]. This data points to 11β-HSD1 as an important component in mediating GC-induced IR in muscles and selective enzyme inhibitors may be among the pharmacologic strategies for the attenuation of such negative GC impacts on insulin-targeting tissues.

Thus, it seems that increased protein phosphorylation of inactivating IRS-1 residues and decreased protein phosphorylation of activating PKB residues seems to be the common factor for the GC-induced impairment in glucose uptake (Figure 1 and Figure 2), especially at resting condition. Whether these responses also occur in a similar magnitude in both gender and different periods of life (young, adult and old) requires further investigation.

## 4. GC Excess and Adipose Tissue: Contribution of Increased Adipose Tissue Lipolysis for GC-Related Glucose Intolerance

Adipose tissue is an endocrine organ that plays a large role in energy provision and storage, predominately mediated by the action of hormones (e.g., insulin, catecholamines, growth hormone, and glucagon) and adipo(cito)kines (e.g., adiponectin, leptin, resistin, tumor necrosis factor (TNF)-α, interleukin (IL)-6, IL-1β). Adipose tissue regulation is also influenced by GCs, although the metabolic effect of this class of hormone on adipose tissue is still somewhat unclear. In Cushing’s syndrome or chronic hypercortisolemia, individuals have increased lipolysis resulting in elevated free fatty acid (FFA) release and altered adipose tissue accumulation and distribution [52] (Figure 1). The marked alterations in adipose distribution is distinct to the Cushing’s phenotype with adipose accumulating in their central (visceral) depots while both skeletal muscle and peripheral adipose tissue get wasted (Figure 1) [53]. These changes may have a negative influence on peripheral insulin sensitivity and glucose metabolism. In addition to altering adipose mass distribution (hypertrophy), GCs contribute to central accumulation through adipose tissue hyperplasia. GCs are well established as a factor for inducing the differentiation of stromal cells to adipocytes, contributing to adipogenesis and de novo lipogenesis, resulting in excessive adiposity [54]. This redistribution and accumulation of body fat centrally and potentially in muscle and liver cells has a significant impact on whole body glucose, lipid and protein metabolism, considering that increased adiposity, specifically at the central depots, is strongly correlated with metabolic syndrome and a marker for insulin resistance [55,56].

As previously discussed, GCs influence glucose metabolism directly by increasing EGP and limiting peripheral glucose disposal. Additionally, they impact glucose metabolism indirectly though altering lipid metabolism. GCs have consistently been shown to increase the rate of lipolysis via increased expression of adipose tissue lipases, which results in the release of glycerol and FFA into the circulation (Figure 1) [57]. Elevated FFAs levels have been seen in both subjects with pharmacologically supraphysiological levels of cortisol (6 h infusion) [58], as well as both in vivo in hypercortisolemic rats (300 mg corticosterone for 10 days) [32] and in vitro in isolated rat adipocytes (DEX for 60 min) [59]. In agreement, short-term treatment of healthy subjects with DEX (2 mg/day for 2 days) results in reduced suppression of FFAs during an insulin clamp [14] (Table 1). Additionally, in vitro adipocyte studies have determined that GCs increase cyclic adenosine monophosphate (cAMP) levels and protein kinase A (PKA) content as well as the mRNA expression of two key lipolytic enzymes, hormone-sensitive lipase and adipose triglyceride lipase [32,59]. Rodent models using Wistar rats implanted with corticosterone pellets (100 mg, for 14 days) have also determined that GC treatment results in reduced AMPK activity in visceral adipose tissue, which further promotes lipolysis, while at the same time increasing lipid storage to promote adipose accumulation [60]. Interestingly, in human adipocytes extracted from obese individuals, DEX (25 nM) has been shown to increase lipoprotein lipase (LPL) activity, which is an antilipolytic enzyme involved in the uptake of FFA into cells and could contribute to adipocyte hypertrophy [61]. In this study, a significantly greater response was seen in adipocytes extracted from the omental versus subcutaneous depot [61]. This was an unexpected finding as typically LPL activity, which is normally stimulated by IGF-1, is higher in subcutaneous versus omental fat. In this model, an additional group of human adipocytes were treated with DEX + insulin (25 nM DEX, 7 nM insulin) and unlike when treated with DEX alone, it was found that elevations in LPL activity were more pronounced in the subcutaneous depot instead of omental, demonstrating the more insulin-resistant state of omental fat. Additionally, when human adipocytes are treated with DEX + insulin, LPL degradation was reduced when compared to treating with insulin alone in both the subcutaneous and omental depots, although the mechanism for this remains unclear [62]. This finding helps to support a potential mechanism for promoting central fat accumulation with increased lipolytic potential. For comprehensive reviews about the actions of GCs on adipose tissue biology and development of central obesity, refer to [63,64]. Once FFAs are taken up, either by adipose tissue or by other metabolic tissues, such as liver or skeletal muscle, they have multiple fates; they may either be oxidized by the mitochondria to be used as a fuel, stored as triglycerides, or converted to a secondary messenger, such as ceramide and diacylglycerol (DAG) [65,66].

In the muscles, elevated circulation of FFAs results in increased intramuscular triglycerides (IMTG) [66], which are associated with the reduction of glucose uptake and are strongly correlated with peripheral IR [66]. In addition to their role in increasing circulating FFAs, GCs may induce IMGT by promoting the differentiation of fibro/adipogenic progenitors into adipocytes within muscle, as seen in a model of mice treated with DEX (1 mg/kg *b.w.* daily for 14 days) [67]. It has previously been hypothesized by Randle et al. [68] that substrates compete to be oxidized through a concept he referred to as the glucose fatty-acid cycle; however, it has since been found that FFA influence glucose sensitivity by interfering with insulin signaling [69]. Accumulation of DAGs within human muscle from obese and T2DM patients results in an increased activation of protein kinase C-θ (PKCθ). This impairs insulin signaling in the muscle through inhibitory phosphorylation of IRS-1 [70], thereby reducing Pi3K and PKB activity, which limits insulin receptor activity, and ultimately impairs insulin-mediated glucose uptake. It is important to note that while studies examining the relation between elevated GCs and DAGs within muscle are lacking, a rodent model using rats implanted with GCs (4 × 100 mg corticosterone) and fed a high-fat diet, termed the “rapid-onset-diabetes” model, found elevated ectopic fat deposition in the muscle of GC-treated rats; this condition was further exacerbated in rats receiving the high-fat diet [71]. However, this data conflicted with a study by Geer and colleagues [53] in which magnetic resonance imaging examinations were performed in Cushing’s patients and healthy controls, and ectopic muscle deposition was not significantly altered between the groups while further studies are required to determine the implications of GC-induced hyperlipidemia on the muscles.

Within liver, hypercortisolism is associated with increased hepatic lipid accumulation and elevated circulation of very low-density lipoprotein and low-density lipoprotein, resulting in elevated circulating triglycerides and cholesterols [72]. Elevated FFAs released from visceral adipose tissue is critical in the development of fatty liver and hepatic steatosis. Additionally, hepatic steatosis is involved in the early development of non-alcoholic fatty liver disease, and is associated with hepatic IR. In a study by Rockall and colleagues [73], hepatic steatosis was prevalent in ~20% of newly diagnosed Cushing’s patients and was positively correlated to visceral fat mass accumulation. This was in agreement with the prior mentioned model of rapid-onset-diabetes in rodents, where elevated GCs resulted in hepatic steatosis [71,74]. These morphological changes in the liver may be due to indirect effects of GCs stimulating lipolysis to elevate circulating FFA levels [75,76], or because of de novo lipogenesis in the liver itself from elevated glucose concentrations [74] (Figure 1). Elevated FFAs have been shown to reduce glycogen breakdown in liver while stimulating gluconeogenesis in an acute fasting state in healthy individuals, resulting in hyperglycemia [77]. In humans, similar results were found in obese but not lean men, in that FFAs contributed to hepatic glycogen retention [78]. In rodents, acutely elevated FFAs increased hepatic lipid accumulation, which increases DAG levels as well as the PKCε membrane translocation. This led to reduced IRS-2 kinase activity, which then impairs Pi3K activity and prevents insulin from inhibiting excess hepatic glucose production [79]. Samuel and Colleagues [80] suggested that PKCε might target the c-Jun N-terminal kinase (JNK)-1 to influence hepatic insulin resistance, although more research is required to determine if there is a connection between the two.

Within adipose tissue itself, GCs seem to play a pleiotropic role on insulin sensitivity, dependent on the fat depot (Figure 1 and Figure 2). Omental human adipocytes, i.e., intra-abdominal adipose or visceral adipose, treated with GCs (0.3 μM DEX, 24 h) have been found to have impaired insulin stimulated glucose uptake. In this study, DEX treatment decreased the content of IRS-1 and PKB protein content in omental but not subcutaneous adipocytes [81]. Additionally, in a rat model of GC-induced IR (1 mg/kg dexamethasone *b.w.* for 5 days), an increase in the epididymal IRS-1 Ser^307^ with reduced PKB Ser^473^ protein phosphorylation was observed after an oGTT in the DEX-treated animals compared to the controls [82]. In agreement with these findings, rodent adipocytes from the epididymal depot, which is comparable to visceral adipose tissue in humans, stimulated with GCs have impaired glucose uptake [13,83] via the down-regulation in the insulin-signaling pathway, specifically PKB [13]. Contrary to this, in humans, overnight infusion of hydrocortisone (0.2 mg/kg/h intravenously) causes increased insulin sensitivity in subcutaneous adipose tissue, despite peripheral insulin resistance [84]. The leading mechanism for these depot specific differences involves the expression of 11β-HSD-1. Adipose-specific 11β-HSD1 over-expression in mice (in subcutaneous and epididymal depots) results in obesity and IR in mice [85], whereas inhibiting 11β-HSD1 enhanced adipose insulin sensitivity [86]. There is an increased expression of 11β-HSD1 in omental versus subcutaneous adipose tissue in humans [87], causing increased GCs bioavailability, which parallels with GCs involvement in impaired insulin sensitivity and increasing adiposity in omental depots, while the subcutaneous adipose depot seems to be protected from these metabolic effects.

Hypercortisolism is also well known for its perturbations in adipokine regulation. Adipokines or adipocytokines are hormones secreted from adipose which influence glucose and lipid metabolism, inflammation, bone remodeling, atherosclerosis, and blood pressure [88]. A reduction in adiponectin levels, a prominent adipokine that positively influences both lipid and glucose metabolism, is commonly associated with IR and obesity [89] and plasma adiponectin levels are reduced with GC exposure in both human [90] and rodent models [91]. Therefore it is likely that reduction of adiponectinemia is an indirect mechanism in which GCs impact on glucose homeostasis. Resistin is another adipokine that impacts on insulin sensitivity in both the liver and in skeletal muscle, but unlike adiponectin, it is associated with reduced insulin sensitivity [92]. Although data examining the interaction of GCs and resistin are scarce, one small study of predominantly females with Cushing’s syndrome found elevated levels of plasma resistin compared to healthy controls which may be an additional factor contributing to the impairments in systemic insulin sensitivity [93]. Additionally, GCs are also known to influence inflammatory cytokines, which may be another way in which GCs indirectly influence systemic glucose metabolism. Both plasma TNF-α and IL-6 are associated with hyperinsulinemia [94] and reduced levels have been found to improved insulin sensitivity. Interestingly, DEX has been shown to decrease both of these cytokines [95,96], which counterbalance some of the impairments GCs induce on insulin signaling.

By this data, it can be noted that GCs excess alters liver and skeletal muscle physiology not only by direct action, but also by indirect, such as with ectopic fat resulting from up-regulated fat lipolysis, or through altered adipo(cyto)kine expression, generally impairing insulin signaling in peripheral tissues. However, more information is required in determining the specific mechanisms by which GC-induced aberrations in lipid metabolism influence systemic glucose homeostasis.

## 5. Effects of GC Excess in the Pancreatic Islets: Contribution of Reduced β-Cell Function for GC-Induced Glucose Intolerance

Pancreatic β-cells exhibit a plastic ability in the regulation of insulin release, but they do so in a precise manner. The prevailing circulating glucose concentration is the main determinant for the quantity of insulin released and while β-cell function remains preserved, blood glucose fluctuates within a relatively narrow physiological range [97]. In healthy individuals, there is a feedback loop between insulin-sensitive tissues and the β-cells in that an increase in peripheral insulin demand is augmented by insulin secretion. Thus, changes in the peripheral insulin sensitivity are reciprocally compensated by changes in circulating insulin levels [98,99]. Failure in this reciprocal loop may result in the disruption of glucose homeostasis and deviations from normal glucose tolerance to the development of glucose intolerance (Figure 1) and, at the extreme, T2DM [97].

As mentioned before, excess GCs alter the insulin sensitivity in insulin-target tissues such as liver, skeletal muscles and adipose tissue. Based on the feedback loop it is expected that increased circulating insulin levels would counteract any alterations in insulin sensitivity imposed by GCs. Notwithstanding, GCs (by direct effects) are known to cause pancreatic β-cell dysfunction as demonstrated in in vitro studies [18,100,101,102]. However, during in vivo GCs exposure, the effects on pancreatic islets are rather indirect and depend on a variety of parameters that include the dose and period of GC regimen, as well as individual susceptibility to GCs [4].

Acutely, GCs cause a negative impact on β-cell function. Healthy individuals receiving 1 mg DEX just prior to an oGTT became glucose intolerant. This was accompanied by a decrease in glucose clearance and unaltered insulin secretion [15]. This data was corroborated by a study conducted with healthy male volunteers subjected to a single dose of 75 mg PRED [17] (Table 1). On the day following PRED treatment, these individuals became glucose intolerant during a meal challenge test and exhibited a relative reduction of β-cell function based on unaltered c-peptide levels with a meal challenge. They also demonstrated impairments in some model-derived parameters that indicate β-cell function (β-cell sensitivity to glucose and potentiation factor ratio, this latter predicts non-glucose potentiating factors) [17]. The mechanisms for the rapid negative effects of GCs on the β-cell function are not yet known in humans and seem to be a direct rather than an indirect effect considering insulin hypersecretion generally compensates for peripheral GC-induced IR. However, it is important to emphasize that 24 h after interruption of PRED administration, all parameters of β-cell function returned to baseline values [17].

For longer periods of GC treatment in humans, for instance, 2–4 days with DEX or 6–15 days with PRED, β-cell function adapts to compensate, at least in parts, to the peripheral IR caused by GCs [9,10,17,31,35]. Then, different degrees of hyperinsulinemia, dependent on the amount of GC exposure, guarantee that blood glucose remain near normal physiologic values (for a comprehensive review, see Rafacho et al. [4]). During a glucose challenge using a hyperglycemic-clamp [9,35,103], or by an oGTT [15,104,105], insulin secretion is higher in healthy subjects treated with GCs (e.g., DEX and PRED) compared to controls, which implies a certain enhancement of β-cell function to compensate for the increased GC-induced peripheral insulin demand. Nonetheless, we cannot rule out that for a more prolonged period these β-cell adjustments may not be enough to retain this peripheral-islet feedback loop.

Various studies led by Diamant’s group have demonstrated that PRED exerts a negative impact on β-cell function. In general, they demonstrated that both at acute and prolonged PRED treatment, with doses varying from 7.5–80 mg (considered low and high doses, respectively) result in the relative impairments of both insulin and c-peptide release in response to a physiologic glucose load, specifically, a meal challenge test [17,19,20]. Several model-derived parameters obtained through plasma insulin, c-peptide, glucagon, and blood glucose values indicate β-cell defects during a meal challenge, which points to the diabetogenic effects of PRED, independent of both dosage and the duration of exposure. From the same group, a study performed by Uyl and colleagues [36] also demonstrated that with certain pathologic conditions, such as early active rheumatoid arthritis (RA) (where GC therapy is crucial for the RA treatment) PRED treatment did ameliorate disease activity without deterioration of glucose tolerance in these patients. According to authors, there is a balance in the diabetogenic and anti-inflammatory GC therapy effects in patients that makes short-term exposure to high dose PRED a beneficial and safe treatment option for most patients [36]. However, corroborating studies that have previously addressed this issue with GCs and RA indicated that GC treatment may significantly increase the incidence of T2DM in RA patients [106]. Thus, individual monitoring is required [36,37,106].

In the same trend, it has been well known for many years that individuals with any degree of susceptibility towards glucose intolerance, yet are still normoglycemic pre-GC treatment, fail to induce adaptive islet compensation that is commonly observed in healthy subjects. These include those with low insulin response to glucose [25] or with low insulin sensitivity [107], obese women [108] and first-degree relatives of patients with T2DM [109]. Thus, in such individuals, GC treatment may disrupt glucose homeostasis and lead to the development of hyperglycemia, reinforcing the point that individual backgrounds must be monitored when employing GC treatment. It is important to highlight that antenatal exposure to high levels of GCs may alter fetal programming in which these individuals become susceptible to metabolic alterations (e.g., less insulin sensitive and glucose intolerant) in adulthood [110,111].

In vitro studies revealed that mechanisms underpinning the direct inhibitory effects of GCs on β-cells’ response to glucose includes defects in upstream oxidative glucose metabolism, intracellular calcium handling, amplifying pathways involving PKA and PKC proteins, as well as the up-regulation of FOXO-1, endoplasmic reticulum dyshomeostasis, and increased generation of reactive oxygen species (for a detailed overview, refer to [4]). These direct negative impacts of GCs on glucose-stimulated insulin secretion (GSIS), as well as the GC-induced β-cell apoptosis are not observed in in vivo normal rodents treated with GCs and will not be addressed in details herein.

Defining potential effects of GCs on β-cells using in vivo models is difficult since systemic metabolic consequences of GC treatments, such as alterations in circulating factors (e.g., glucose, FFA, hormones), likely mask the GC-mediated changes in β-cell function. Additionally, isolation of islets from humans is not fully available for most of laboratories, which have directed most of mechanistic aspects of GCs on islet function to rodent models. The main findings to date, (see Figure 2), are summarized herein. Islets isolated from GC-treated rodents (1 mg/kg, *b.w.* for 5 days) have augmented glucose responsiveness [16,112,113,114], higher glucose sensitivity (lower EC_50_ values to glucose) [16], and exhibit pronounced first and second phase GSIS compared to saline-treated animals [16,18] (Table 1). The components underpinning this insulin hypersecretion involve the enhancement of glucose sensitivity with stimulus secretion coupling (e.g., increased islet nicotinamide adenine dinucleotide phosphate generation, and enhanced mitochondrial function and intracellular calcium in response to glucose) [115]. In addition, the amplification of the pathway of GSIS, which involves the activation of calcium-dependent kinases, is up-regulated in DEX-treated rat islets [115,116]. Rats treated with 1 mg DEX for 5 days have high islet content of phosphorylated PKCα, which parallels the augmented number of docked insulin-containing granules in β-cells, as well as increased insulin secretion in response to PKC activator phorbol-12-myristate 13-acetate [115]. The higher β-cell function in DEX-treated rats also involves up-regulation of cholinergic signals [16,115,117], non-glucidic insulin secretagogues (amino acids and FFA) [16,118], non-metabolic signals [116], cAMP-dependent protein kinases [116], insulin signaling proteins (e.g., phosphorylated insulin receptor β, IRS-2, Pi3K, p-PKB, phosphorylated PKB substrate (p-AS160)) [115,119], and augmented content of the gap junction intercellular communication protein connexin 36 [114,120]. In addition, increased β-cell mass is developed after short-term GC exposure in rats and mice [18,33,114]. Almost all of these adaptive islet compensations are age- and sex- [121], dose- [33], and time-dependent [18], being progressively larger relative to the reciprocal decrease in peripheral insulin sensitivity. Altogether, this demonstrates that with an acute period (no more than 5 days) of DEX treatment, the peripheral-islet loop (the disposition index) seems to be relatively preserved, resulting in insulin hypersecretion that may or may not avoid glucose intolerance and elevation in fasting blood glucose. More recently, it was demonstrated that rodents, mainly rats, treated with DEX (1 mg/kg *b.w.* for 5 days) have reduced insulin clearance and lower hepatic activity of insulin-degrading enzyme, which may also corroborate for the hyperinsulinemia in these animals [122].

The scenario where GC excess results in in vivo impairment of β-cell function is better observed in susceptible animals (e.g., *Zucker* rats and *ob/ob* mice). It was shown that *Zucker* rats exposed to high doses of DEX develop significant insulinopenia [123]. In the same trend, obese mice treated with 25 μg DEX for 1–2.5 days (at a dose equivalent to 0.5 mg/kg *b.w.*) exhibit diminished or inhibited GSIS [124,125]. The mechanisms by which this incidence of insulinopenia occurs may involve α2-adrenergic signals [126]. Based on this data, it is possible to speculate that, as in humans, rodents develop insulinopenia depending on their previous glucose homeostasis vulnerability before introduction of GCs; reinforcing that although caution is required for administration of GCs in healthy subjects, those with already deteriorating glucose intermediate metabolism require special individual monitoring.

In addition to the relatively poor insulin secretion in response to certain experimental contexts of GC excess, elevations in circulating glucagon levels may also be found both in humans [20,103,127], rhesus macaques [128], and in rodent models [40,129]. Adult rats treated with DEX for 5 days at 1 mg/kg, *b.w.*, exhibit glucose intolerance and insulin resistance that parallels with increased α-cell mass, higher glucagonemia and impaired inhibition of glucagon release at high glucose concentration (11.1 mM) [40]. Considering the hyperglycemic action of glucagon on glucose metabolism, and that impaired glucose homeostasis is partially attenuated after administration of an antagonist of glucagon receptor (in a rat model of GC-induced hyperglycemia), it is important to take into account the role of α-cell dysfunction on the diabetogenic actions of GCs. For a comprehensive review on the non-pancreatic β-cells hormones readers are invited to read [30].

## 6. Conclusions

GCs are undoubtedly amongst the hormones with pleiotropic actions. GCs constitute a class of steroid hormones with important therapeutic applications in disorders involving inflammatory, allergic, and immunologic responses. Despite their efficacy as an anti-inflammatory and immunosuppressive agent, GCs’ side effects are not negligible. The knowledge concerning GCs’ potential and limitations in clinical practice have permitted the use of safe treatments, when taking into account their potential adverse effects. However, some patients cannot always be free of GC side effects and, among them, glucose intolerance may prevail. The knowledge about GCs’ implications on glucose metabolism is well known, but the molecular mechanisms by which GCs affect such tissues have not been fully elucidated. For instance, how much do the adverse effects depend on genomic or non-genomic GC actions? Although it seems difficult to translate the findings from animals to humans, many aspects are reproducible among them (e.g., GC-induced glucose intolerance), which made these rodent models suitable for mechanistic studies. The cross-talk between peripheral tissues involved in the control of glucose metabolism is becoming even more consistent in the way of scientific knowledge advances, and GCs affect all of these peripheral tissues. In this sense, which tissues are more or less affected? And which tissue is affected by GCs’ therapies first? What about these adverse effects in repetitive treatments? These questions merit further investigation. There is also preclinical evidence pointing to the fact that GC-mediated side effects on metabolism are partially dependent on endocannabinoids, through peripheral endocannabinoid receptor 1 [130], illustrating how far we are from full elucidation of GC mechanisms of action. Another aspect to be investigated is regarding why some patients are more or less responsive to GC therapies and/or to GC side-effects? Previous metabolic susceptibility such as that observed in obese, metabolic syndrome and T2DM patients must be taken into account. Accordingly, how much we know about the metabolic susceptibilities carried by maternal and/or paternal influences? Such concerns should also be encouraged in studies involving GCs. While alternative drugs with expected pharmacological GC actions, without major side effects, are not yet a reality, it is important to consider the benefits of GCs and include the practice of a healthy lifestyle (e.g., regular physical exercises, fed health and functional diets) as an alternative supporting counterbalance of GC side effects.

## Figures and Tables

**Table 1:** Metabolic repercussions of GC treatment in human and rats.

	Study Design	Hepatic Glucose Output	Skeletal Muscle Glucose Uptake	Adipose Tissue Lipolysis	Glycemia and Insulinemia	β-cell Function	Ref.
HUMAN	DEX (4 × 0.5 mg) for 2 days – women	Increased	Unaltered	Unaltered	Normal glycemia and increased insulinemia	Increased	[9]
	PRED (7.5 and 30 mg) for 14 days – men	Increased (both doses)	Unaltered	Increased (both doses)	Increased glycemia (both doses) and insulinaemia (higher dose)		[10]
	DEX (2 mg) for 2 days – both genders	Unaltered	Reduced	Probably increased	Increased insulinaemia, but not glycemia		[11]
	DEX (4 × 0.5 mg) for 2 days – men	Increased	Reduced	Increased			[14]
	DEX (4 × 0.5 mg) for 2 days – both genders	Unaltered	Probably reduced		Unaltered glycemia Increased insulinemia		[15]
	PRED (75 mg) for 1 day or (30 mg) for 15 days – men					Reduced for 75 mg, but unaltered for 30 mg treatment (based on plasma C-peptide)	[17]
	DEX (15 mg) over 3 days - women				Unaltered glycemia Increased insulinemia	Increased	[31]
RAT	DEX (1 mg) for 7 days – male Wistar rats	Increased			Increased glycemia and insulinemia		[6]
	DEX (0.5 mg) for 7 days – male Wistar rats	Increased	Reduced		Increased glycemia and insulinaemia		[8]
	DEX (1 mg) for 11 days – male Wistar rats		Reduced	Increased	Unaltered glycemia		[13]
	CORT (300 MG) wax pellets for 10 days – male SD rats			Increased	Unaltered insulinemia		[32]
	DEX (1 mg) for 5 days – male Wistar rats				Increased glycemia and insulinemia		[18,33]

Read Section 2, Section 3, Section 4 and Section 5 for complete details. **CORT;** corticosterone, **DEX;** dexamethasone**, PRED;** prednisolone, **SD;** Sprague-Dawley, **Ref.;** reference.

## Abbreviations

AMPK	5′ Adenosine Monophosphate-Activated Kinase
cAMP	cyclic Adenosine Monophosphate
DAG	Diacylglycerol
DEX	Dexamethasone
EGP	Endogenous Glucose Production
FFA	Free Fatty Acids
FoxO-1	Forkhead Box O-1
GC	Glucocorticoid
GLUT-4	Glucose Transporter 4
GR	Glucocorticoid Receptor
GRdim	Genetic Engineered Mice Model of GR Dimerization
GS	Glycogen Synthase
GSIS	Glucose-Stimulated insulin Secretion
GSK-3	Glycogen Synthase Kinase 3
G6Pase	Glucose-6-Phosphatase
HGO	Hepatic Glucose Output
HGP	Hepatic Glucose Production
IGF-1	Insulin-Like Growth Factor 1
IL	Interleukine
IMTG	increased intramuscular triglycerides
IR	Insulin Resistance
IRS-1	Insulin Receptor Substrate 1
IRS-2	Insulin Receptor Substrate 2
KK	mouse model of metabolic syndrome
LPL	Lipoprotein Lipase
LXR	Liver X Receptors
MKP-3	MAP kinase phosphatase 3
mRNA	messenger Ribonucleic Acid
oGTT	oral Glucose Tolerance Test
PEPCK	Phosphoenolpyruvate-Carboxykinase
Pi3K	Phosphoinositide 3-Kinase
PKA	Protein Kinase A
PKB	Protein Kinase B
PKC	Protein Kinase C
PP-1	Protein Phosphatase 1
p-PKB	Phosphorylated Protein Kinase B
PRED	Prednisolone
RA	Rheumatoid Arthritis
RXR	Retinoid X Receptors
TNF-α	Tumor Necrosis Factor α
T2DM	Type 2 Diabetes *Mellitus*
2-[^3^H]DG	2-[^3^H] Deoxyglucose
11β-HSD-1	11β Hydroxysteroid Dehydrogenase Type 1
11-DHC	11 Dehydrocorticosterone
